# Supplementary material for: Mechanisms governing the pioneering and redistribution capabilities of the non-classical pioneer PU.1
Source: Nat Commun. 2020 Jan 21;11:402. doi: 10.1038/s41467-019-13960-2 (PMC6972792; doi:10.1038/s41467-019-13960-2)
Supplement: Supplementary file 7 — Source data [file 41467_2019_13960_MOESM7_ESM.zip › Source_Data/Figure6/Figure6B_MotifScanOutput/ETS1_mutPU.1/homerResults/motif21.similar.html]

motif21

## Information for motif21

G
A
T
C
C
G
T
A
C
T
A
G
G
T
A
C
G
A
C
T
A
C
T
G
C
G
A
T
A
G
T
C
C
G
T
A
  
Reverse Opposite:  

G
C
A
T
T
C
A
G
G
C
T
A
A
G
T
C
C
T
G
A
A
C
T
G
G
A
T
C
C
G
A
T
C
T
A
G
  

|  |  |
| --- | --- |
| p-value: | 1e-110 |
| log p-value: | -2.536e+02 |
| Information Content per bp: | 1.665 |
| Number of Target Sequences with motif | 3783.0 |
| Percentage of Target Sequences with motif | 28.05% |
| Number of Background Sequences with motif | 6528.6 |
| Percentage of Background Sequences with motif | 18.61% |
| Average Position of motif in Targets | 98.7 +/- 56.1bp |
| Average Position of motif in Background | 98.9 +/- 63.1bp |
| Strand Bias (log2 ratio + to - strand density) | 0.0 |
| Multiplicity (# of sites on avg that occur together) | 1.17 |
| Motif File: | file (matrix) reverse opposite |

### Similar de novo motifs found

|  |  |  |  |  |  |  |  |
| --- | --- | --- | --- | --- | --- | --- | --- |
| Rank | Match Score | Redundant Motif | P-value | log P-value | % of Targets | % of Background | Motif file |
| 1 | 0.859 | C G T A T A C G A G T C C G T A C T A G G T A C G C A T T C A G A G C T A G T C C G A T | 1e-54 | -124.343935 | 9.12% | 5.17% | motif file (matrix) |
| 2 | 0.846 | T G A C C G A T A T C G C T G A A G T C C G T A A C T G A G C T A C G T A C T G C T G A | 1e-47 | -110.081944 | 10.12% | 6.16% | motif file (matrix) |
| 3 | 0.832 | G T C A G T C A T C A G A G T C G C T A C A T G G T A C C G A T A C T G A C G T A G T C C G A T | 1e-47 | -108.375874 | 7.03% | 3.80% | motif file (matrix) |
| 4 | 0.876 | C A T G A G T C C G T A C T A G G A T C C G A T A C T G | 1e-43 | -101.136700 | 26.30% | 20.35% | motif file (matrix) |
